# Supplementary material for: Century Wide Changes in Macronutrient Levels in Indian Mothers’ Milk: A Systematic Review
Source: Nutrients. 2022 Mar 27;14(7):1395. doi: 10.3390/nu14071395 (PMC9002949; doi:10.3390/nu14071395)
Supplement: Supplementary file 1 [file nutrients-14-01395-s001.zip › 7. Supplementary Table S1.pdf]

Table S1. Profile of the records and key findings included in the review (n = 34).

	First Author, Year	Type of Study	n, Maternal Age	Macronutrients Studied	Location	SES	Time Of Sample Collection, Type of Milk	Lactational Stage	Estimation Method/s Used			Key Study Findings	Quality Score (Rating)	Ref.
									Fat	Protein	Lactose			
**1**	Bunce, 1931	Cross-sectional	8 (3 Indian mothers), age NA	Fat, protein, lactose	Burma (currently Myanmar)	NA	NA, NA	Mature	Soxhlet	NA	NA	Macronutrient content of milk from Burmese, Indian, Anglo Indian, and European mothers is comparable	0 (0)	[62]
**2**	Sundararajan, 1941	Case Control	35 for protein, 40 for lactose, age range 18–38 years. Data for mothers without Beriberi was considered	Protein, lactose	Coonoor, Tamil Nadu, South India	Low	Morning -by manual expression and few by breast pump < NA	Mature (in two groups -Infant < 4 months and Infants > 4 months)	Soxhlet	Biuret	Benedict Quantitative Assessment	There is no difference in protein and lactose content of milk of mothers whose children are below 4 months or above 4 months of age.	8 (i)	[63]
**3**	Srinivasan, 1954	Case Control	25 healthy mothers, 6 mothers with children suffering from kwashiorkar, age NA. Data for healthy mothers was considered	Protein	Coonoor, Tamil Nadu, South India	Low	NA, NA	Mature (1 month to two years)	NA	Kjeldahl	NA	No significant difference in protein content of milk between apparently healthy mothers and mothers with children suffering from Kwashiorkar.	8 (i)	[64]
**4**	Karmarkar, 1958	Cross-sectional	175, age NA	Fat	Baroda, Gujarat, West India	Comparison of SES groups	Morning -by manual expression and few by breast pump, NA	Mature (not reported, considered mature)	Chiba et al	NA	NA	Milk of mothers belonging to very poor SES contains significantly less fat than the other three (poor, middle, upper-middle). The increase in dietary fat up to a certain amount may increase the fat in breastmilk	8 (i)	[65]

**5**	Gopalan P1, 1958	Case series	6, 20 – 30 years	Fat, protein, lactose	Coonoor, Tamil Nadu, South India	Low	24-hour sampling, NA	Mature (66th to 100th weeks)	NA	NA	NA	Protein content of milk tended to decline slightly in the later stages of lactation	7 (i)	[43]
**6**	Gopalan P2, 1958	Cross-sectional	40, age NA	Fat, protein, lactose	Coonoor, Tamil Nadu, South India	Low	24-hour sampling, NA	Mature (4-24 weeks of lactation)	NA	NA	NA	Mean value of protein (1·06 ± 0·036g/100ml), lactose (7·47 ±0·072 g/100ml) and fat 3·34± 0·242 g/100ml) in 40 mothers between 4-24 weeks of lactation	6 (i)	[43]
**7**	Belavady, 1959	Cross-sectional	29, age NA	Protein	Coonoor, Tamil Nadu, South India	Low	Morning -by manual expression and few by breast pump, NA	Mature (<1 month and >1 month)	NA	Kjeldahl	NA	Protein content of milk fell after 1st month of lactation; remained constant almost thereafter.	8 (i)	[66]
**8**	Belavady, 1959	Cross-sectional	36 for protein, 20 for fat and lactose, age NA	Fat, protein, lactose	Madras, Tamil Nadu, South India	Low	Morning -by manual expression and few by breast pump, NA	Mature (1-36 months)	Soxhlet	Kjeldahl	By calculation	Chemical composition of breast milk of mothers belonging to three tribes concerning the proximate principles was found to be similar to that of the poor South Indian women.	8 (i)	[67]
**9**	Belavady, 1959	Cross-sectional	191, age NA	Fat, protein, lactose	Coonoor, Tamil Nadu, South India	Low	Morning -by manual expression and few by breast pump, NA	Colostrum (1st-2nd day), transitional (3rd-10th day) and mature (11 days-18 months)	Soxhlet	Kjeldahl	By calculation	The content of protein, fat and lactose were higher in first month as compared to other stages of lactation. The proximate principles in breast milk of poor Indian women were comparable with the reported values from	8 (i)	[55]

												other countries with better diets.		
**10**	Mukherji, 1959	Case Control	35, age NA	Protein	Nagpur, Maharashtra, Central India	Low	Morning- between 10-11 am, Foremilk	Mature (2nd month- 5 years)	NA	Kjeldahl	NA	Colostrum contains a large percentage of protein which gradually diminishes during the next few weeks. Age of infant, mother and parity didn't affect the chemical composition of milk. However, protein content in the breastmilk was found to be slightly higher in mothers on mixed feeding than those on complete vegetarian diet.	9 (i)	[68]
**11**	Sinha, 1959	Cross-sectional	63, 17-35 years	Fat, protein, lactose	Patna, Bihar, East India	Low & middle class	Morning- two samples, 15 ml milk before and after the baby had suckled the breast and were mixed together, NA	Colostrum (<7days) and mature milk (3rd-40th week)	Babcocks	Kjeldahl	Bock's	The blood protein, haemoglobin levels of the mother, the economic condition of mother and the time-lapse postpartum, variation in diet did not seem to significantly influence the composition of milk.	8 (i)	[69]
**12**	Karmarkar, 1959	Cross-Sectional	232, age NA	Fat, protein, lactose	Baroda, Gujarat, West India	Different SES	Afternoon between two feeds at about 3 p.m., NA	Mature milk (0-12 months)	Chiba et al	Kjeldahl	Chiba et al	A sharp decline in the values for fat and protein, observed after the first month, while no change is observed in the values for lactose. The milk obtained from very poor is found to contain significantly less fat	9 (i)	[70]

											than that obtained from the other three groups (t=2.862) whereas for protein and lactose it is comparable		
**13**	Karmarkar, 1960	Cohort	60, age NA	Fat, protein, lactose	Baroda, Gujarat, West India	Low	Afternoon between two feeds at about 3 p.m., NA	Mature milk (3-4 months)	Chiba et al	Kjeldahl	Chiba et al	Increase in dietary protein (r =0·3733, t = 3·253, p <0·05) and fat (r=0·7932, t = 2·128, p < 0·05) up to a certain level had a favourable effect on milk values, increase beyond that level had no effect. No effect was seen in lactose content of milk	8 (i)	[71]
**14**	Ashdhir, 1962	Cross-sectional	10, 19-34 years	Protein, lactose	Delhi, North India	Middle and High	Morning -by breast pump and few by manual expression, NA	Colostrum (3rd day), transitional (8th day) and mature (18th day)	NA	Kjeldahl	Hawk, Oser & Summerson	Lactose increases and protein decreases slightly with progression in lactation stages. There was a positive correlation between dietary intake and breast milk constituents, but it was NS for protein (r=0·047, t = 0·133, p < 0·05) and lactose (r=0·214, t = 0·6183, p < 0·05).	9 (i)	[72]
**15**	Deb, 1962	Case series	20, age NA	Fat, protein, lactose	Bangalore, Karnataka, South India	Low	Morning, NA	Mature (2-10 months)	Hawk, Oser & Summerson	Kjeldahl	Hawk, Oser & Summerson	Dietary supplementation with protein did not appear to increase the total protein concentration of the milk but the quality	8 (ii)	[73]

												of the milk protein was changed		
**16**	Karmarkar, 1963	Cross-sectional	60 (5 in control group), age NA	Fat, protein	Baroda, Gujarat, West India	Low	Morning, Foremilk	Mature (1st-3rd month)	Chiba et al	Kjeldahl	NA	The fat and protein contents of milk increase to a ceiling level with the dose supplemented till dietary levels of 50-55 g are reached in regard to either.	10 (i)	[74]
**17**	Khurana, 1970	Cross-sectional	194, age NA	Protein	Delhi, North India	Different SES groups	NA, NA	Colostrum (<5 days), transitional (6-15 days) and mature (16days-9 months)	NA	Kjeldahl	NA	The protein content was observed to be higher in colostrum as compared to transitional and mature milk ($p<0\cdot125>0\cdot01$). There was no significant difference observed in milk composition of mothers belonging to different SES groups.	8 (i)	[75]
**18**	Jathar, 1970	Cross-sectional	48, 18-35 years	Protein	Mumbai, Maharashtra, West India	Low	Morning before first feed, NA	Not mentioned, considered mature milk	NA	Biuret	NA	NS difference in protein levels between various dietetic groups- lactovegetarian, non-vegetarian (occasional meat eaters) and non-vegetarians (frequent meat eaters)	8 (i)	[76]
**19**	Rao, 1973	Cross-sectional	31, age NA	Protein	Hyderabad, Telangana, South India	NA	NA, NA	Colostrum (2-5 days), mature (1-12 months)	NA	Acid extraction and Sephadex gel filtration	NA	The protein content of colostrum was 2·5± 0·.54g /100ml and 1·0± 0·04 g/ 100ml in mature milk	2 (0)	[77]

**20**	Agarwal, 1975	Cross-sectional	97, age NA	Protein	Varanasi, Uttar Pradesh, North India	NA	NA, NA	Colostrum (<5 days), transitional (6-15 days) and mature (>16days)	NA	Kjeldahl	NA	The total protein content in colostrum was significantly higher than transitional and mature milk (p<0·001).	4 (0)	[78]
**21**	Belavady, 1978	Cross-sectional	NA	Fat	Hyderabad, Telangana, South India	Low	Morning, NA	Mature milk (<1 month-12 months)	Folch & associates	NA	NA	The concentration of lipid and the fatty acid composition did not differ with duration of lactation, except in colostrum.	1 (0)	[79]
**22**	Rao, 1981	Cross-sectional	70, Age NA	Fat, protein	Hyderabad, Telangana, South India	Low	Morning, NA	Colostrum (<5 days), transitional (6-15 days) and mature (16days-12 months)	Folch & associates	Kjeldahl	NA	Total lipid concentration remained essentially unaltered with the duration of lactation (p<0·02)	7 (i)	[80]
**23**	Bijur, 1985	Case Control	50, 18-38 years	Protein	Bombay, Maharashtra, West India	Low	Morning before first feed, NA	Not mentioned, considered mature milk	NA	Biuret	NA	No significant difference (p<0·05) in protein levels between various dietetic groups- lactovegetarian, non-vegetarian (occasional meat eaters) and non-vegetarians (frequent meat eaters)	7 (i)	[81]
**24**	Kumbhat, 1985	Cross-sectional	50, 18–40 years (majority between 21–25 yrs)	Fat, protein, lactose	Mumbai, Maharashtra, West India	Low & Middle	Morning- between 10-11 am, NA	Colostrum (5th day), transitional (10th-15th day) and mature (20th-25th day)	Sulphuric acid method	Kjeldahl	Copper Reduction	Protein content was higher initially and then declined by 25th day post-partum. Protein content was higher in the breastmilk of mothers who delivered prematurely. Protein content was	10 (i)	[82]

												significantly higher in middle income group than low-income group (p<0·05). Fat content didn't alter with gestation but was found to be slightly higher in high SES group.		
**25**	Raghuvanshi, 1988	Cohort	121, 15–38 years	Protein	Varanasi, Uttar Pradesh, North India	NA	Morning, milk sample during second feed, NA	Colostrum (3-7 days), transitional (8-15 days) and mature (>15days-15 months)	NA	Kjeldahl	NA	The total protein was significantly higher in colostrum as compared to mature milk (p<0·05). The well-nourished women showed marginally higher means for protein. Women hemoglobin didn't show any difference for breast milk parameters.	10 (i)	[83]
**26**	Garg, 1988	Cross-sectional	35, 18-30 years	Fat, protein	Delhi, North India	High and Low	Morning before first feed, NA	Colostrum (0-3 days)	Colorimetry	Colorimetry	NA	The protein content in colostrum of the well-nourished group (6·04 ± 0·73 g/ dl) was significantly higher than poorly nourished group (4·5± 1·2g/dl) p<0·05. The total fat content in the colostrum of the well-nourished mothers (7·72 g/dl) was almost double than that in the colostrum of the under-nourished group (3·79 g/dl)	9 (i)	[58]

**27**	Patil, 1989	Cross-sectional	54, age NA	Fat, protein, lactose	Mumbai, Maharashtra, West India	High	Morning, NA	Transitional (5-10 days)	Chromic acid method	Lowry method	Folin-Wu Method	The fat (4·48 ±1·50 g/100ml), protein (1·08 ±0·42 g/100ml) and lactose (6·51 ±1·28 g/100ml) content of breastmilk of well-nourished Indian women was comparable with well-nourished women of developed countries.	6 (i)	[84]
**28**	Paul, 1997	Cohort	52 (23 term and 29 pre-term infants) data for term infants was considered, age NA	Fat, protein, lactose	Delhi, North India	NA	NA, Foremilk	Colostrum (3rd day), transitional (7th and 14th day), and mature (21st day)	Waku kit	Lowry method	Standard Nelson Somogyi Method.	Differences in milk composition between preterm and term infants was NS. There was a decline in protein content with increasing post-partum age while fat and lactose content increased	9 (i)	[85]
**29**	Kaushik, 2002	Case Control	80, data for 20 control group mothers were considered, 18 – 30 yrs (mean age 22 yrs)	Fat, protein, lactose	Delhi, North India	NA	NA, NA	Colostrum (Within 24 hours of delivery)	Colorimetr	Lowry method	Folin-Wu Method	Medical complications during pregnancy like diabetes (n=20), PIH (n=20), anemia (n=20) etc can affect the composition of colostrum. There was a significant difference between protein content in milk of mothers with PIH ($p<0·01$) and anemia ($p<0·05$), lactose and fat with diabetes ($p<0·01$) as compared to control group (n=20).	9 (i)	[86]

**30**	Narang, 2006	Cohort	86, 21-35 yrs, Data for 41 term infants included	Fat, protein, lactose	Ludhiana, Punjab, North India	NA	NA, NA	Colostrum (3rd day), transitional (7th and 14th day), and mature (21st day)	Milkoscan	Milkoscan	Milkoscan	Fat and lactose in preterm milk was significantly lower than term milk ($p<0{\cdot}01$). Fat and lactose were found to increase in amount with increasing postnatal age. Protein levels were observed to be significantly higher in preterm than term milk ($p<0{\cdot}01$). These were observed to decrease significantly with increase in postnatal age.	11 (ii)	[87]
**31**	Roy, 2013	Cross-sectional	217, age NA	Fat	Kolkatta, West Bengal, East India	Different SES groups	Morning, Hindmilk	Mature	Folch method	NA	NA	BMI was linearly proportional to total lipids. Total lipid in breast milk was significantly lower in Low SES as compared to medium and high SES ($p<0{\cdot}05$)	9 (i)	[49]
**32**	Dias, 2016	Cross-sectional	63, Mean age 28 yrs;	Protein, lactose	Sion, Mumbai, Maharashtra, West India	NA	Morning, NA	Colostrum (3rd day)	NA	Biuret	Benedict's test	No statistically significant difference in lactose of breastmilk of mothers classified based on BMI (under, normal, over-nourished). The protein was significantly lower in undernourished mothers when compared with both normal ($p<0{\cdot}015$) and over-nourished mothers ($p<0{\cdot}001$).	7 (i)	[88]

**33**	Kothari, 2018	Cross-sectional	63, Mean age 28 yrs	Protein, lactose	Sion, Mumbai, Maharashtra, West India	NA	Morning, NA	Colostrum (3rd day)	NA	Biuret	Benedict Quantitative Assessment	No statistically significant difference in lactose in colostrum of mothers classified based on BMI. Protein in colostrum was significantly lower in undernourished mothers as compared to normal and over-nourished mothers	8 (i)	[89]
**34**	Divedi, 2020	Case Control	132, Non-anemic (n=66) mean age: 25·12±4·12 years; anemic (n=66) mean age: 25·27±3·32 years. Data for non-anemic mothers included	Fat, protein, lactose	Etawah, Uttar Pradesh, North India	NA	Morning after one hour of previous breastfeeding, NA	Transitional (4th-11th day)	Milk analyzer	Milk analyzer	Milk analyzer	Severe and moderate anaemia causes significant changes in fat, lactose, and protein content of breast milk ($p<0·0001$), while mild anaemia has some effect on lactose and protein contents but not on fat	8 (i)	[90]

NA: Not Available; SES: Socioeconomic status; NS: Not significant
Quality was rated as 0 for poor (0–4 out of 14 questions), i for fair (5–10 out of 14 questions), or ii for good (11–14 out of 14 questions)
