# Supplementary material for: Century Wide Changes in Macronutrient Levels in Indian Mothers’ Milk: A Systematic Review
Source: Nutrients. 2022 Mar 27;14(7):1395. doi: 10.3390/nu14071395 (PMC9002949; doi:10.3390/nu14071395)
Supplement: Supplementary file 1 [file nutrients-14-01395-s001.zip › 8. Supplementary Table S2.pdf]

Supplementary Table 2: QUALITY ASSESSMENT OF STUDIES INCLUDED IN THE REVIEW

Table 2.1: Quality assessment for Cross-sectional & cohort studies (n=22) included in the review

AUTHOR/S, YEAR OF PUBLICATION	STUDY	1 Was the research question or objective in this paper clearly stated?	2 Was the study population clearly specified and defined?	3 Was the participation rate of eligible persons at least 50%?	4 Were all the subjects selected or recruited from the same or similar populations (including the same time period)? Were inclusion and exclusion criteria for being in the study prespecified and applied uniformly to all participants?	5 Was a sample size justification, power description, or variance and effect estimates provided?	6 For the analyses in this paper, were the exposure(s) of interest measured prior to the outcome(s) being measured?	7 Was the timeframe sufficient so that one could reasonably expect to see an association between exposure and outcome if it existed?	8 For exposures that can vary in amount or level, did the study examine different levels of the exposure as related to the outcome (e.g., categories of exposure, or exposure measured as continuous variable)?	9 Were the exposure measures (independent variables) clearly defined, valid, reliable, and implemented consistently across all study participants?	10 Was the exposure(s) assessed more than once over time?	11 Were the outcome measures (dependent variables) clearly defined, valid, reliable, and implemented consistently across all study participants?	12 Were the outcome assessors blinded to the exposure status of participants?	13 Was loss to follow-up after baseline 20% or less?	14 Were key potential confounding variables measured and adjusted statistically for their impact on the relationship between exposure(s) and outcome(s)?	Overall score	Overall rating
Bunce1931	Cross sectional	X	X	X	X	X	NA	NA	NA	NA	NA	NA	NA	NA	NA	0	0
Gopalan 1958 (P2)	Cross sectional	1	1	X	1	X	X	1	X	1	X	1	X	NA	X	6	i
Karmarkar et al 1958	Cross sectional	1	1	X	1	X	1	1	1	1	X	1	X	X	X	8	i
Belavady 1959	Cross sectional	1	1	X	1	X	1	1	1	1	X	1	X	X	X	8	i
Belavady, Pasricha, Shankar1959	Cross sectional	1	1	X	1	X	1	1	1	1	X	1	X	NA	X	8	i

Belavady & Gopalan1959	Cross sectional	1	1	X	1	X	1	1	1	1	X	1	X	NA	X	8	i
Sinha et al 1959	Cross sectional	1	1	X	1	X	1	1	1	1	X	1	X	NA	X	8	i
Karmarkar et al 1959	Cross sectional	1	1	NR	1	X	1	1	1	1	X	1	X	NR	1	9	i
Karmarkar & Ramakrishnan 1960	Cohort	1	1	X	1	X	1	1	1	1	X	1	X	NA	X	8	i
Ashdhir & Puri 1960	Cross sectional	1	1	X	1	X	1	1	1	1	X	1	X	NA	1	9	i
Karmarkar et al 1963	Cross sectional	1	1	X	1	X	1	1	1	1	1	1	X	NA	1	10	i
Khurana et al 1970	Cross sectional	1	1	X	1	X	1	1	1	1	X	1	X	X	X	8	i
Jathar et al 1970	Cross sectional	1	1	X	1	X	1	1	1	1	X	1	X	NA	X	8	i
Rao & Belavady 1973	Cross sectional	1	X	NA	NA	X	NA	NA	X	X	X	1	NA	NA	X	2	0
Agarwal et al 1975	Cross sectional	1	NA	X	NA	X	NA	NA	1	1	X	1	NA	NA	X	4	0
Belavady 1978	Review article: Cross sectional	X	1	NA	NA	NA	NA	NA	NA	NA	NA	X	NA	NA	NA	1	0
Rao & Belavady 1981	Cross sectional	1	1	X	NA	X	1	1	1	1	X	1	X	NA	X	7	i
Kumbhat et al 1985	Cross sectional	1	1	X	1	X	1	1	1	1	1	1	X	NA	1	10	i
Raghuvanshi et al 1988	Cohort	1	1	X	1	X	1	1	1	1	1	1	X	CD	1	10	i
Garg et al 1988	Cross sectional	1	1	X	1	X	1	1	1	1	X	1	X	NA	1	9	i
Patil & Phadke 1989	Cross sectional	1	1	X	1	X	1	NR	NR	1	X	1	X	NA	X	6	i

Paul et al 1997	Cohort	1	X	X	X	X	1	1	1	1	1	1	X	1	1	9	i
Narang et al 2006	Cohort	1	1	X	1	X	1	1	1	1	1	1	X	1	1	11	ii
Roy et al 2013	Cross sectional	1	1	X	1	X	1	1	1	1	X	1	X	NA	1	9	i
Dias & Nakhawa 2016	Cross sectional	1	X	NR	1	X	1	NA	1	1	X	1	X	NA	1	7	i
Kothari et al 2018	Cross sectional	1	1	X	1	NA	1	NA	1	1	X	1	NA	NA	1	8	i

*CD, cannot determine; NA, not applicable; NR, not reported;
Quality was rated as 0 for poor (0–4 out of 14 questions), i for fair (5–10 out of 14 questions), or ii for good (11–14 out of 14 questions)

Table 2.2: Quality assessment for Case-control studies (n=6) included in the review

	1	2	3	4	5	6	7	8	9	10	11	12		

	Was the research question or objective in this paper clearly stated and appropriate?	Was the study population clearly specified and defined?	Did the authors include a sample size justification?	Were controls selected or recruited from the same or similar population that gave rise to the cases (including the same timeframe)?	Were the definitions, inclusion and exclusion criteria, algorithms or processes used to identify or select cases and controls valid, reliable, and implemented consistently across all study participants?	Were the cases clearly defined and differentiated from controls?	If less than 100 percent of eligible cases and/or controls were selected for the study, were the cases and/or controls randomly selected from those eligible?	Was there use of concurrent controls?	Were the investigators able to confirm that the exposure/risk occurred prior to the development of the condition or event that defined a participant as a case?	Were the measures of exposure/risk clearly defined, valid, reliable, and implemented consistently (including the same time period) across all study participants?	Were the assessors of exposure/risk blinded to the case or control status of participants?	Were key potential confounding variables measured and adjusted statistically in the analyses? If matching was used, did the investigators account for matching during study analysis?	Overall score	Overall rating
Sundararajan 1941	1	1	X	1	1	1	NR	1	1	1	X	X	8	i
Srinivasan & Ramanathan 1954	1	1	X	1	1	1	NR	1	1	1	X	X	8	i
Mukherji & Anwikar 1959	1	1	X	1	1	1	NR	1	1	1	X	1	9	i
Bijur & Desai 1985	1	1	X	1	1	1	X	CD	1	1	X	X	7	i
Kaushik et al 2002	1	1	X	1	1	1	X	1	1	1	X	1	9	i

Divedi et al 2020	1	1		1	1	1	X	CD	1	1	X	1	8	i

*CD, cannot determine; NA, not applicable; NR, not reported.
Quality was rated as 0 for poor (0–4 out of 12 questions), i for fair (5–9 out of 12 questions), or ii for good (10–12 out of 12 questions)

Table 2. 3: Quality assessment for Case-Series studies (n=2) included in the review

		Gopalan 1958 (P1)	Deb & Cama, 1962
1	Was the study question or objective clearly stated?	1	1
2	Was the study population clearly and fully described, including a case definition?	1	1
3	Were the cases consecutive?	1	1
4	Were the subjects comparable?	1	1
5	Was the intervention clearly described?	NA	1
6	Were the outcome measures clearly defined, valid, reliable, and implemented consistently across all study participants?	1	1
7	Was the length of follow-up adequate?	1	1
8	Were the statistical methods well-described?	X	X
9	Were the results well-described?	1	1
	Overall score	**7**	**8**
	Overall rating	**i**	**ii**

*CD, cannot determine; NA, not applicable; NR, not reported;
Quality was rated as 0 for poor (0–3 out of 9 questions), i for fair (4–7 out of 9 questions), or ii for good (8–9 out of 9 questions);
